# Risk factors associated with cytomegalovirus reactivation in patients receiving immunosuppressive therapy for rheumatic diseases: a retrospective study

**DOI:** 10.1038/s41598-022-25451-4

**Published:** 2022-12-03

**Authors:** Tatsuya Shimada, Misako Higashida-Konishi, Keisuke Izumi, Satoshi Hama, Tatsuhiro Oshige, Hisaji Oshima, Yutaka Okano

**Affiliations:** 1 Division of Rheumatology, Department of Medicine, National Hospital Organization Tokyo Medical Center, Tokyo, Japan; 2 Division of Rheumatology, Department of Internal Medicine, Keio University School of Medicine, Tokyo, 1608582 Japan

**Keywords:** Rheumatic diseases, Rheumatology, Risk factors

## Abstract

Immunosuppressive treatment is a common cause of cytomegalovirus (CMV) reactivation. However, there is no consensus regarding the risk factors for CMV reactivation in rheumatic diseases. Therefore, this study aimed to elucidate the risk factors associated with CMV reactivation. We retrospectively collected the data of 472 patients with rheumatic diseases whose CMV pp65 antigen (C7-HRP) titer was measured. We divided the patients into those with and those without C7-HRP. We retrospectively collected data on age, sex, primary condition and organ involvement, and blood test results. We also investigated the use of immunosuppressants and the maximum and cumulative doses of prednisolone (PSL). We performed univariate and multivariate analyses to identify risk factors for CMV reactivation. Multivariate analysis showed that higher age (71.2 vs. 64.4 years, p = 0.0022), hypoalbuminemia (2.9 vs. 3.4 g/dL, p = 0.0104), higher creatinine level (1.2 vs. 0.9 mg/dL, p = 0.0026), cyclosporine use (8.2 vs. 3.6%, p = 0.0101), and higher maximum (552.4 vs. 243.3 mg, p < 0.0001) and cumulative (2785.9 vs. 1330.5 mg, p < 0.0001) doses of PSL were associated with CMV reactivation. Older age, hypoalbuminemia, higher creatinine level, cyclosporine use, and higher maximum and cumulative doses of PSL were significant risk factors for CMV reactivation in rheumatic diseases.

## Introduction

Reactivation of cytomegalovirus (CMV) is an adverse event reported in immunocompromised patients^1^. Rheumatic diseases frequently require immunosuppressive treatment. Previous studies have reported several risk factors of CMV reactivation, including male sex, elderly age, disease activity or severity, lymphocytopenia, hypoalbuminemia, elevated liver enzyme and creatinine levels, cyclophosphamide and cyclosporine use, and prednisolone (PSL) dose^2,3^. However, strong evidence is unavailable, primarily because of the small number of cases. Therefore, we investigated the characteristics of CMV-positive and CMV-negative patients receiving treatment for rheumatic diseases to identify the risk factors for CMV reactivation.

## Methods

### Study design

The study was approved by the institutional review committee of the National Hospital Organization Tokyo Medical Center (approval number: R20-181). The need for written informed consent from patients was waived according to the regulations in Japan and the National Hospital Organization Tokyo Medical Center.

### Inclusion criteria

We retrospectively evaluated the data of consecutive patients with rheumatic diseases who were admitted to our department between January 2006 and October 2021. Next, we identified patients with measurements of CMV pp65 antigen (C7-HRP) titer.

### Exclusion criteria

Patients who had been followed up in our department for less than 6 months were excluded.

### Data collection

We classified the patients into two groups: patients with C7-HRP (CMV-positive) and those without the antigen (CMV-negative). The positive or negative diagnosis depended on the initial result of positivity or negativity during the patients’ consultation with our department. C7-HRP titer was measured commercially by SRL (Tokyo, Japan). We collected data on age, sex, mean values of blood test results within 3 months before C7-HRP titer measurement, primary condition, and organ involvement. We also investigated the use of immunosuppressants and the maximum and cumulative doses of PSL administered within 3 months before C7-HRP titer measurement. The maximum and cumulative doses of PSL contained a methylprednisolone pulse that was converted into a PSL equivalent: 1000 mg of methylprednisolone was converted into 1250 mg of PSL, for example. Further, we classified patients with CMV-positive disease into CMV-treated and CMV-untreated patients: the former were treated with anti-CMV drug and the latter were not. Anti-CMV treatment included either ganciclovir, valganciclovir, and phosphonomethanoic acid. We collected blood test results at the start of treatment for CMV activation to estimate the reason for treating CMV reactivation.

### Statistical analyses

Objective variables were the positivity for C7-HRP and use of anti-CMV drug. Explanatory variables were age, sex, primary condition, organ involvement, and mean values of blood test results. Concerning quantitative variables, outcomes with Gaussian distribution showed median from 25 to 75 percentiles. On the other hand, outcomes without Gaussian distribution showed average plus or minus standard deviation. We performed multivariate analysis of the explanatory variables. Student’s t-test or Pearson's chi-square test was performed for bivariate analyses. Multivariate analyses were performed using Pearson’s chi-square test. A p-value of < 0.05 was considered statistically significant. Statistical analyses were performed using JMP version 16.1.0 (SAS Institute, Cary, NC, USA).

### Ethics approval and consent to participate

This study was approved by the institutional review committee of the National Hospital Organization Tokyo Medical Center (approval number: R20-181), and the written informed consent from the patients was waived according to the regulations in the National Hospital Organization Tokyo Medical Center.

### Criteria, guidelines, and regulations

All rheumatic diseases were diagnosed using the relevant criteria (Supplementary Table 1). All methods were performed in accordance with the relevant guidelines and regulations.

## Results

### Patient disposition

**Figure 1:**
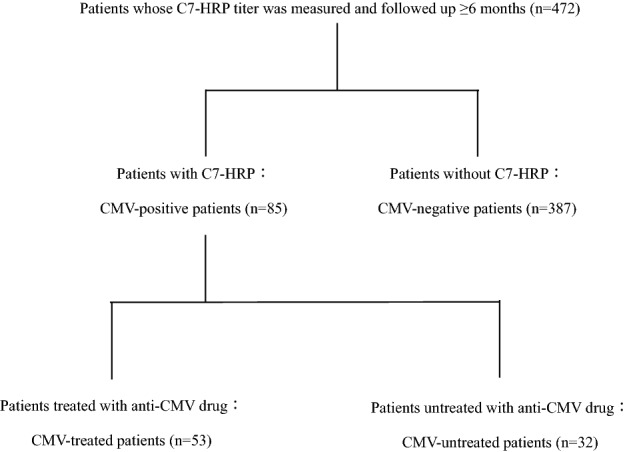
Classification of patients.

Figure 1 shows the flowchart of patient disposition. All patients who received ≥ 1 mg/kg PSL were subjected to C7-HRP titer measurements.

### Baseline characteristics and primary conditions

Baseline characteristics (age and sex) and primary conditions of the patients are summarized in Table 1. The overall population of our patients was 472, and all of them were Asian. Of the 472 patients included in the study, 85 tested positive and 387 tested negative for C7-HRP. The average age of patients in CMV-positive and CMV-negative groups was 71.2 and 64.4 years, respectively (p = 0.0021). The male-to-female ratio was 20/65 and 120/267, respectively (p = 0.0290). No significant difference was observed between the initial administration of PSL and C7-HRP titer measurement (263.5 vs. 535.8 days, p = 0.2228).

**Table 1:** Baseline characteristics, common primary problem, and blood test results.

	CMV-positive patients (n = 85)	CMV-negative patients (n = 387)	p value
**Baseline characteristics and common primary problem**			
Age, mean ± SD, years	71.2 ± 15.1	64.4 ± 18.4	0.0022
Female, n (%)	65 (76.4%)	267 (69.0%)	0.0290
Duration between maximum dose of PSL and C7-HRP measurement, mean ± SD, days	263.5 ± 944.6	535.8 ± 1360.0	0.2228
Microscopic polyangiitis, n (%)	18 (21.2%)	15 (3.9%)	< 0.0001
Rheumatoid arthritis, n (%)	16 (18.8%)	131 (33.9%)	0.0009
Systemic lupus erythematosus, n (%)	14 (16.5%)	53 (13.7%)	0.0761
Adult-onset Still’s disease, n (%)	6 (7.1%)	5 (1.3%)	0.0002
Dermatomyositis, n (%)	4 (4.7%)	17 (4.4%)	0.1026
Eosinophilic granulomatosis with polyangiitis, n (%)	4 (4.7%)	12 (3.1%)	0.0706
Systemic sclerosis, n (%)	4 (4.7%)	8 (2.1%)	0.0273
Sjogren’s syndrome, n (%)	2 (2.4%)	23 (5.9%)	0.0305
Takayasu arteritis, n (%)	2 (2.4%)	7 (1.8%)	0.0959
Granulomatosis with polyangiitis, n (%)	2 (2.4%)	4 (1.0%)	0.0530
Anti-synthetase syndrome, n (%)	2 (2.4%)	4 (1.0%)	0.0530
Mixed connective tissue disease, n (%)	2 (2.4%)	4 (1.0%)	0.0530
Polymyalgia rheumatica, n (%)	1 (1.2%)	27 (7.0%)	0.0065
Proliferative nephritis, n (%)	1 (1.2%)	13 (3.4%)	0.0467
Behcet’s disease, n (%)	1 (1.2%)	13 (3.4%)	0.0467
Giant cell arteritis, n (%)	1 (1.2%)	9 (2.3%)	0.0759
IgG4-related disease, n (%)	1 (1.2%)	7 (1.8%)	0.0921
Polymyositis, n (%)	1 (1.2%)	4 (1.0%)	0.1027
Malignant rheumatoid arthritis, n (%)	1 (1.2%)	2 (0.5%)	0.0740
**Blood test results**			
White blood cell count, mean ± SD, /µL	9260 ± 5190	8580 ± 4900	0.0001
Neutrophil count, mean ± SD, /μL	7720 ± 4660	6440 ± 4040	0.0001
Lymphocyte count, mean ± SD, /μL	820 ± 660	1190 ± 980	< 0.0001
Hemoglobin level, mean ± SD, g/dL	10.4 ± 2.1	11.1 ± 2.3	< 0.0001
Platelet count, mean ± SD, /μL	233,000 ± 125,000	259,000 ± 119,000	0.0002
Albumin level, mean ± SD, g/dL	2.9 ± 0.6	3.4 ± 0.8	0.0104
Creatinine level, mean ± SD, mg/dL	1.2 ± 1.0	0.9 ± 0.6	0.0026

The relatively common diseases in the patients were rheumatoid arthritis (n = 147), systemic lupus erythematosus (SLE; n = 67), microscopic polyangiitis (n = 33), Sjogren’s syndrome (n = 25), and dermatomyositis (n = 21). Microscopic polyangiitis (21.2 vs. 3.9%, p < 0.0001) was significantly more common in the CMV-positive group than in the CMV-negative group. Rheumatoid arthritis (18.8 vs. 33.9%, p = 0.0009) and Sjogren’s syndrome (2.4 vs. 3.5%, p = 0.0305) were significantly more common in the CMV-negative group than in the CMV-positive group.

### Blood test results

Blood test results for each patient are presented in Table 1. Average neutrophil counts (7720 vs. 6440/μL, p = 0.0001) and creatinine level (1.2 vs. 0.9 mg/dL, p = 0.0026) were significantly higher in the CMV-positive group than in the CMV-negative group. In contrast, hemoglobin level (10.1 vs. 11.1 g/dL, p < 0.0001), lymphocyte count (820 vs. 1190/μL, p < 0.0001), platelet count (233,000 vs. 259,000/μL, p = 0.0002), and albumin level (2.9 vs. 3.4 g/dL, p = 0.0104) were significantly lower in the CMV-positive group than in the CMV-negative group.

### Organ involvement of primary conditions

Common organ involvement in the primary condition is listed in Table 2. The most common comorbidities in the CMV-positive group were interstitial lung disease (35.3 vs. 16.0%, p < 0.0001), nephritis (23.5 vs. 11.6%, p = 0.0005), peripheral nervous system disorders (11.8 vs. 5.7%, p = 0.0070), alveolar hemorrhage (5.9 vs. 0.8%, p = 0.0001), and peripheral circulatory disorders (4.7 vs. 1.6%, p = 0.0111). Arthritis was the most common comorbidity in all patients (n = 203); however, its frequency was significantly lower in the CMV-positive group than in the CMV-negative group (18.8 vs. 48.3%, p < 0.0001).

**Table 2:** Organ involvement of primary problem.

	CMV-positive patients (n = 85)	CMV-negative patients (n = 387)	p value
Interstitial lung disease, n (%)	30 (35.3%)	62 (16.0%)	< 0.0001
Nephritis, n (%)	20 (23.5%)	45 (11.6%)	0.0005
Arthritis, n (%)	16 (18.8%)	187 (48.3%)	< 0.0001
Peripheral nerve disorders, n (%)	10 (11.8%)	22 (5.7%)	0.0070
Rash, n (%)	8 (9.4%)	34 (8.8%)	0.1013
Myositis, n (%)	7 (8.2%)	28 (7.2%)	0.0965
Pancytopenia, n (%)	6 (7.1%)	16 (4.1%)	0.0412
Fever, n (%)	5 (5.9%)	11 (2.8%)	0.0272
Alveolar hemorrhage, n (%)	5 (5.9%)	3 (0.8%)	0.0001
Peripheral circulatory disorders, n (%)	4 (4.7%)	6 (1.6%)	0.0111
Pleuritis, n (%)	3 (3.5%)	22 (5.7%)	0.0660
Sore throat, n (%)	3 (3.5%)	6 (1.6%)	0.0380
Eosinophilia, n (%)	3 (3.5%)	6 (1.6%)	0.0380
Pericarditis, n (%)	3 (1.9%)	6 (1.6%)	0.0380
Pulmonary hypertension, n (%)	3 (3.5%)	1 (0.3%)	0.0004
Optic neuritis, n (%)	3 (3.5%)	0 (0.0%)	< 0.0001
Lymphadenopathy, n (%)	2 (2.4%)	7 (1.8%)	0.0959
Central nervous system lupus, n (%)	2 (2.4%)	4 (1.0%)	0.0530
Meningitis, n (%)	2 (2.4%)	4 (1.0%)	0.0530
Pneumocystis pneumounia, n (%)	2 (2.4%)	2 (0.5%)	0.0158
Genital ulcer, n (%)	2 (2.4%)	1 (0.3%)	0.0842
Gastrontestinal ulcer, n (%)	2 (2.4%)	1 (0.3%)	0.0043
Peritonitis, n (%)	2 (2.4%)	1 (0.3%)	0.0043
Myelitis, n (%)	2 (2.4%)	1 (0.3%)	0.0043
Epicarditis, n (%)	1 (1.2%)	1 (0.3%)	0.0398
Oral aphtha, n (%)	1 (1.2%)	7 (1.8%)	0.0921
Enteritis, n (%)	1 (1.2%)	3 (0.8%)	0.0944

### Medication

Immunosuppressive therapies administered to each patient are listed in Table 3. Higher maximum doses of PSL (552.4 vs. 243.3 mg/day, p < 0.0001), intravenous cyclophosphamide (27.1 vs. 11.4%, p < 0.0001), rituximab (9.4 vs. 2.1%, p < 0.0001), azathioprine (23.5 vs. 14.2%, p = 0.0053), and cyclosporine (8.2 vs. 3.6%, p = 0.0101) were more frequently administered in the CMV-positive group than in the CMV-negative group. The average cumulative doses of PSL were 2785.9 vs. 1330.5 mg in the CMV-positive and CMV-negative groups, respectively (p < 0.0001).

**Table 3:** Medication.

	CMV-positive patients (n = 85)	CMV-negative patients (n = 387)	p value
Maximum dose of PSL, mean ± SD, mg/day	552.4 ± 621.1	243.3 ± 510.4	< 0.0001
Cumulative amounts of PSL, mean ± SD, mg	2785.9 ± 2018.8	1330.5 ± 1611.1	< 0.0001
Intravenous cyclophosphamide, n (%)	23 (27.1%)	44 (11.4%)	< 0.0001
Azathioprine, n (%)	20 (23.5%)	55 (14.2%)	0.0053
Tacrolimus, n (%)	19 (22.4%)	81 (20.9%)	0.0977
Methotrexate, n (%)	13 (15.3%)	112 (28.9%)	0.0014
Salazosulfapyridine, n (%)	8 (9.4%)	92 (23.8%)	0.0004
Rituximab, n (%)	8 (9.4%)	8 (2.1%)	< 0.0001
Cyclosporine, n (%)	7 (8.2%)	14 (3.6%)	0.0101
Mizoribine, n (%)	6 (7.1%)	34 (8.8%)	0.0858
Bucillamine, n (%)	3 (3.5%)	36 (9.3%)	0.0133
Iguratimod, n (%)	3 (3.5%)	14 (3.6%)	0.1036
Mycophenolate Mofetil, n (%)	1 (1.2%)	14 (3.6%)	0.0409
Sarilumab, n (%)	1 (1.2%)	6 (1.6%)	0.0989
Sodium aurothiomalate, n (%)	1 (1.2%)	5 (1.3%)	0.1032
Baricitinib, n (%)	1 (1.2%)	3 (0.8%)	0.0944

### Multivariate analysis between CMV-positive and CMV-negative groups

We performed multivariate analysis with patient age, albumin level, creatinine level, cyclophosphamide and cyclosporine use, and maximum and cumulative doses of PSL as variables (Table 4). These variables were selected because they showed significance in univariate analysis and have also been reported to be associated with CMV reactivation in previous studies^1–5^. The results of the present study suggest that older age (p = 0.0076), albumin level (p < 0.0001), creatinine level (p = 0.0140), cyclosporine use (p = 0.0229), maximum dose of PSL (p = 0.0009), and cumulative dose of PSL (p < 0.0001) were significantly associated with CMV positivity.

**Table 4:** Multivariate analysis of risk factors associated with cytomegalovirus pp65 antigen positivity.

	Odds ratio	95% CI	p value
Age	1.030	1.008–1.052	0.0076
Female ratio	0.642	0.299–1.380	0.1357
Albumin level	0.224	0.121–0.414	< 0.0001
Creatinine level	1.623	1.103–2.387	0.0140
Maximum dose of PSL	0.997	0.996–0.999	0.0009
Cumulative amount of PSL	1.001	1.001–1.002	< 0.0001
Intravenous cyclophosphamide	1.383	0.591–3.236	0.4550
Cyclosporine	4.187	1.291–14.379	0.0229

### Comparison between CMV-treated and CMV-untreated disease groups

Baseline characteristics (age and sex) and latest blood test results of the CMV-treated and CMV-untreated disease groups at the start of treatment against CMV activation are listed in Table 5. Anti-CMV drugs were administered to 63.5% of patients in the CMV-positive group. The average age was higher of patients who received the anti-CMV drug treatment than of those who did not (73.7 vs. 67.1 years, p = 0.0492). The corresponding male-to-female ratio was 40/13 vs. 25/7 (p < 0.0001). Blood test results were not significantly different between the CMV-treated and CMV-untreated disease groups.

**Table 5:** Characteristics of CMV-treated and CMV-untreated patients.

	CMV-treated patients (n = 53)	CMV-untreated patients (n = 32)	p value
Age, mean ± SD, years	73.7 ± 11.5	67.1 ± 19.1	0.0492
Female, n (%)	40 (75.5%)	25 (78.1%)	< 0.0001
Neutrophil count, mean ± SD, /μL	7116 ± 4102	6154 ± 2915	0.2525
Lymphocyte count, mean ± SD, /μL	640 ± 492	708 ± 549	0.5596
Hemoglobin level, mean ± SD, g/dL	10.7 ± 1.9	10.6 ± 2.3	0.8380
Platelet count, mean ± SD, /μL	181,000 ± 148,000	182,000 ± 87,000	0.9610
C-reactive protein level, mean ± SD, mg/dL	2.7 ± 4.7	2.9 ± 4.4	0.8406
Aspartate aminotransferase level, mean ± SD, IU/L	36.6 ± 54.4	24.8 ± 13.0	0.2318
Alanine aminotransferase level, mean ± SD, IU/L	50.9 ± 89.3	32.2 ± 30.4	0.2537
Creatinine level, mean ± SD, mg/dL	0.9 ± 0.8	1.1 ± 1.2	0.4516
Immunoglobulin G level, mean ± SD, mg/dL	1192 ± 906.4	976 ± 308.2	0.5249

## Discussion

Studies have regarded older age as a risk factor for CMV reactivation that includes rheumatic diseases and overall immunosuppressive conditions^6^. A decrease in the count of naïve T cells and an increase in that of aged CD8 T cells may be involved in the compromise of CMV in the elderly^7–9^. The present study also revealed that older age was associated with CMV reactivation. Moreover, female sex was associated with CMV reactivation in univariate analysis, but there is no consensus on the association between sex and CMV reactivation in previous studies.

Concerning blood test results, several studies reported that symptomatic CMV reactivation is associated with hypoalbuminemia^2,10^. Elevated creatinine level was also thought to have positive impacts on CMV reactivation^3,5^. Our study supported the possibility that hypoalbuminemia and elevated creatinine levels could be risk factors for CMV reactivation not only in univariate but also in multivariate analysis.

Regarding primary conditions, Fujimoto et al. reported that patients with SLE, polymyositis, and dermatomyositis were significantly susceptible to CMV reactivation^5^. Pulmonary and renal involvement are major problems of these rheumatic diseases; therefore, high doses of PSL, including methylprednisolone, are preferred^11–13^. Cyclophosphamide and rituximab are also used for remission induction^14–17^. Our study revealed that microscopic polyangiitis, interstitial lung disease, and nephritis were significantly more frequent in CMV-positive patients, who might receive intensive immunosuppressive treatment.

Corticosteroid administration is a major concern for the immunocompromised status. Corticosteroids suppress the activation of lymphocytes, especially CD4 T lymphocytes, that may affect susceptibility to CMV reactivation^18–20^. Previous reports mentioned that higher dose of PSL was especially influential in CMV reactivation^2–5,10,21^. However, regarding detailed usage, especially about initial dose and PSL duration, these opinions remain controversial. Our study is meaningful for two meanings: First, we focused on the initial maximum and cumulative doses of PSL among 472 patients. Second, multivariate analysis also showed significance of PSL dose between CMV-positive and CMV-negative patients.

Other immunosuppressive drugs may also have the same impact on the immunological status. Cyclophosphamide and rituximab mainly inhibit B-lymphocyte activation, but they may also suppress CD4 T lymphocyte activation^22–26^. Cyclosporine may reduce CD4 T lymphocyte count by inhibiting mitogen-activated protein kinase signaling pathways^27,28^. Univariate analysis revealed significance of these immunosuppressants, which may also be the cause of CMV reactivation.

Concerning the past CMV infection, a previous report mentioned that CMV-specific immunoglobulin G (CMV-IgG) level was not significantly related to CMV reactivation^21^. Our study also showed no significance of CMV-IgG positivity (Supplementary Table 2).

The present study has several limitations. First, the study included all rheumatic diseases that complicated the assessment of the severity and activity of each disease. A few previous studies have mentioned them, but one previous study on antineutrophil cytoplasmic antibody-associated vasculitis suggested a connection between the Birmingham vasculitis activity score and CMV reactivation^29^. Disease severity and activity may directly be linked to patients’ physical status and treatment; therefore, unbiased management of multiple rheumatic diseases should be considered for further assessment of CMV reactivation.

Second, the present study was biased toward severe cases that required intensive immunosuppressive treatment. Microscopic polyangiitis was particularly common in CMV-positive patients in the study, probably because it often presents with life-threatening symptoms, such as pulmonary and renal lesions, that require long-term hospitalization. We believe that doctors frequently measure C7-HRP levels in hospitalized patients, whereas they seldom measure it in outpatients without severe symptoms. Therefore, to reduce confounding factors between problems and medication, routine C7-HRP measurements in all outpatients and inpatients will be desirable.

Finally, few CMV-positive had organ involvement, which might complicate doctors’ decisions to prescribe anti-CMV drugs. Only two CMV-positive patients presented with clear symptoms: one with colitis and one with pneumonia. There is currently no consensus on the necessity of anti-CMV drugs; therefore, medication tend to depend on symptoms and blood test results^30–32^. Approximately 30% of CMV reactivation cases are symptomatic, and a majority involve cytopenia^33–36^. However, various factors, including the primary condition, medication, and infection, may affect the complete blood count, making it difficult to identify the direct factor contributing to CMV reactivation. Thus, to assess the necessity of anti-CMV drugs, more cases of symptomatic CMV reactivation and long-term follow-up of blood test results are required.

To improve generalizability of our results, validations in other institutes will be desirable.

## Conclusion

Older age, hypoalbuminemia, elevated creatinine level, cyclosporine use, and higher maximum and cumulative doses of PSL, may be associated with CMV reactivation. A large population, prospective, cohort study is required to address all rheumatic diseases and their impact on CMV reactivation.

## Supplementary Information

Supplementary Tables.

## Data Availability

The data sets used and/or analyzed during the current study are not publicly available for privacy reasons, but are available from the corresponding author on reasonable request.
