# Supplementary material for: Risk factors associated with cytomegalovirus reactivation in patients receiving immunosuppressive therapy for rheumatic diseases: a retrospective study
Source: Sci Rep. 2022 Dec 3;12:20926. doi: 10.1038/s41598-022-25451-4 (PMC9719476; doi:10.1038/s41598-022-25451-4)
Supplement: Supplementary file 1 — Supplementary Tables. [file 41598_2022_25451_MOESM1_ESM.docx]

Supplementary information

**Risk factors associated with cytomegalovirus reactivation in patients receiving immunosuppressive therapy for rheumatic diseases: a retrospective study**

Tatsuya Shimada^1, 2^, Misako Higashida-Konishi^1^, Keisuke Izumi^1, 2,*^, Satoshi Hama^1^, Tatsuhiro Oshige^1, 2^, Hisaji Oshima^1^, and Yutaka Okano^1^

^1^ Division of Rheumatology, Department of Medicine, National Hospital Organization Tokyo Medical Center, Tokyo, Japan

^2^ Division of Rheumatology, Department of Internal Medicine, Keio University School of Medicine, Tokyo, Japan

*Corresponding author: Keisuke Izumi

Division of Rheumatology, Department of Internal Medicine, Keio University School of Medicine, 1608582, Tokyo, Japan.

Tel: +81-3-5363-3786, Fax: +81-3-5379-5037, Email: [izz@keio.jp](mailto:izz@keio.jp)

| **Supplementary table 1. Diagnostic or classification criteria of rheumatic diseases.** | |
| --- | --- |
| Disease | Diagnositic or classification criteria |
| Microscopic polyangiitis | MHLW (1998) |
| Rheumatoid arthritis | ACR (1987), ACR/EULAR (2010) |
| Systemic lupus erythematosus | ACR (1997), SLICC (2012) |
| Adult-onset Still’s Disease | Yamaguchi criteria (1992) |
| Dermatomyositis | MHLW (2014) |
| Eosinophilic granulomatosis with polyangiitis | Lanham criteria (1984), ACR (1990), MHLW (1998) |
| Systemic sclerosis | MHLW (2003), ACR/EULAR (2013) |
| Sjogren’s syndrome | MHLW (1999), ACR (2012) |
| Takayasu Arteritis | ACR (1990), MHLW (2006) |
| Granulomatosis with Polyangiitis | ACR (1990) |
| Anti-synthetase syndrome | MHLW (2014) |
| Mixed connective tissue disease | MHLW (2004) |
| Polymyalgia rheumatica | ACR/EULAR (2012) |
| Proliferative nephritis | ACR (1990), MHLW (2006) |
| Behcet’s disease | MHLW (2010) |
| Giant cell arteritis | ACR (1990) |
| IgG4-related disease | MHLW (2010) |
| Polymyositis | MHLW (2014) |
| Malignant rheumatoid arthritis | MHLW (1998) |

MHLW: Ministry of Health, Labour and Welfare of Japan, ACR: American College of Rheumatology, EULAR: European Alliance of Associations for Rheumatology, SLICC: Systemic Lupus International Collaborating Clinics.

| **Supplementary table 2. Comparison of CMV reactivation rates with and without CMV-IgG.** | | |
| --- | --- | --- |
|  | C7-HRP positive, n (%) | C7-HRP negative, n (%) |
| CMV-IgG positive (n=114) | 28 (24.6%) | 86 (75.4%) |
| CMV-IgG negative (n=14) | 1 (7.1%) | 13 (92.9%) |

Pearson’s chi-square test: p=0.1417.
